# Beta adrenergic blockade reduces utilitarian judgement

**DOI:** 10.1016/j.biopsycho.2012.09.005

**Published:** 2013-02

**Authors:** Terbeck Sylvia, Kahane Guy, McTavish Sarah, Savulescu Julian, Levy Neil, Hewstone Miles, Philip J. Cowen

**Affiliations:** a University of Oxford, Department of Experimental Psychology, South Parks Road, Oxford OX1 3UD, England, United Kingdom; b Univerisity of Oxford, Department of Psychiatry, Neurosciences Division, Warneford Hospital, Oxford OX3 7JX, United Kingdom; c University of Oxford, Oxford Uehiro Centre for Practical Ethics Suite 8, Littlegate House, St. Ebbes Street, Oxford OX1 1PT, United Kingdom

**Keywords:** Noradrenaline, Propranolol, Moral decision-making, Utilitarian judgement

## Abstract

Noradrenergic pathways are involved in mediating the central and peripheral effects of physiological arousal. The aim of the present study was to investigate the role of noradrenergic transmission in moral decision-making. We studied the effects in healthy volunteers of propranolol (a noradrenergic beta-adrenoceptor antagonist) on moral judgement in a set of moral dilemmas pitting utilitarian outcomes (e.g., saving five lives) against highly aversive harmful actions (e.g., killing an innocent person) in a double-blind, placebo-controlled, parallel group design. Propranolol (40 mg orally) significantly reduced heart rate, but had no effect on self-reported mood. Importantly, propranolol made participants more likely to judge harmful actions as morally unacceptable, but only in dilemmas where harms were ‘up close and personal’. In addition, longer response times for such personal dilemmas were only found for the placebo group. Finally, judgments in personal dilemmas by the propranolol group were more decisive. These findings indicate that noradrenergic pathways play a role in responses to moral dilemmas, in line with recent work implicating emotion in moral decision-making. However, contrary to current theorising, these findings also suggest that aversion to harming is not driven by emotional arousal. Our findings are also of significant practical interest given that propranolol is a widely used drug in different settings, and is currently being considered as a potential treatment for post-traumatic stress disorder in military and rescue service personnel.

## Highlights

► We investigated the role of noradrenergic transmission in moral decision-making. ► The beta-adrenoceptor antagonist propranolol affected responses to moral dilemmas. ► Propranolol reduced the willingness to endorse utilitarian solutions involving harm. ► Propranolol also reduced heart rate and response times, and increased decisiveness. ► Blockade of noradrenergic arousal appears to increase aversion to harming others.

## 1 Introduction

Propranolol is a beta-adrenoceptor antagonist that suppresses noradrenergic activation by blocking beta 1 and beta 2 adrenoreceptors. These receptors are located in target areas of the peripheral sympathetic nervous system, as well as in various brain regions including the amydgala (Chamberlain et al., 2006). Several studies have shown that single doses of propranolol influence emotional processing (Hurlemann et al., 2010; Van Stegeren et al., 2005), and that propranolol reduces physiological markers of high arousal (heart rate, potentiated startle response) following emotion-evoking stimuli (Davis et al., 1993; Mills and Dimsdale, 1991). In addition, functional neuroimaging studies have shown that propranolol leads to a reduction in amygdala responses to highly emotional pictures or emotional facial expressions (Hurlemann et al., 2010; Strange and Dolan, 2004; Van Stegeren et al., 2005).

Although propranolol was originally developed for hypertension, its inhibitory effect on the physiological aspects of emotional arousal also makes it an effective and widely used treatment for stress, acute anxiety, and performance anxiety (e.g., Tyrer and Lader, 1974; Weatley, 1969). Recent research has suggested that, by reducing the consolidation of emotional memory, propranolol might also be an effective treatment for post-traumatic stress disorder (PTSD), whether by being administered before exposure to a potentially traumatic situation or immediately afterwards (Mills and Dimsdale, 1991; Pitmann et al., 2002; Stein et al., 2007; Vaiva et al., 2003). The possibility that propranolol could be used in this way in the context of military or rescue operations raises serious ethical concerns (Craigie, 2007; Wolfendale, 2008). For example, Henry et al. (2007) speculated that propranolol might, by decreasing anxiety, alter practical decisions in war, such as assessment of danger or of others’ needs. The President's Council on Bioethics (2003, p. 224) warned that propranolol might diminish decision quality by reducing the physiological arousal that normally guides certain acts. However, the possible influence of propranolol on moral decision-making has not yet been studied.

The present study aimed to investigate the relation between propranolol and moral decision-making. This relation is of considerable practical interest, but it might also shed new light on an important theoretical debate about the role of emotion in moral decision-making (Huebner et al., 2008). Recent research has suggested that moral judgments are often based on immediate emotion-laden intuitions or gut reactions, with little input from reasoning (Haidt, 2001). A central strand of research in this area has investigated responses to hypothetical ‘personal’ moral dilemmas in which, in order to save several lives, it is necessary to directly harm or even kill an innocent person. Functional neuroimaging studies have reported that responses to such ‘personal’ dilemmas are associated with increased activation in brain areas implicated in emotional processing, such as the ventromedial prefrontal cortex (VMPC) and amygdala, compared to responses to ‘impersonal’ dilemmas where the harm is less direct (Greene et al., 2001, 2004). It has been argued that ‘deontological’ moral judgments opposing personal harm, even at the cost of a greater number of lives lost, are based on a pre-potent emotional aversion to harming others, but that this emotional response can sometimes be overcome with effort, leading to a more reason-based utilitarian response (Greene et al., 2004; Greene, 2008). In line with this hypothesis, patients with damage to the VMPC exhibit increased utilitarian judgement in response to high conflict personal dilemmas compared to healthy individuals, a finding which has been attributed to a deficit in social emotion (Ciaramelli et al., 2007; Koenigs et al., 2007). In addition, Moretto et al. (2010) report that healthy individuals, but not patients with VMPC damage, generated a skin conductance response (SCR), a somatic marker of affective arousal, before making utilitarian judgments in personal dilemmas. Moreover, this anticipatory SCR was negatively correlated with the frequency of such utilitarian judgments in healthy individuals.

This research suggests that aversion to harm in personal moral dilemmas is at least partly mediated by emotional arousal. Since propranolol has been consistently shown to reduce noradrenergic mediated physiological arousal, it offers a means to investigate the role of such arousal in moral judgement. We hypothesised that if deontological judgments against personal harm are based on an immediate negative affective response then propranolol, compared to placebo, should lead to an increase in utilitarian judgement (Levy, 2007). The present study was designed to assess this hypothesis by investigating the effect of a single dose of propranolol on moral decision-making in healthy volunteers.

## 2 Method

### 2.1 Participants

Forty participants (20 males/20 females; mean age: 24.3; 34 were students) were recruited via poster and newspaper advertisement. Participants were instructed to refrain from alcohol or coffee 24 h before the study. Full written consent was obtained from all participants and the study was approved by the central National Health Service ethics committee.

### 2.2 Procedure

Individuals were first screened to exclude those with any medical contraindication for propranolol (e.g., asthma, low blood pressure). Participants with any past or present Axis 1 Psychiatric Disorder, as measured using the Structured Clinical Interview for DSM-IV (SCID-IV), were also excluded.

Propranolol (40 mg) or placebo was administrated in identical capsules in a parallel group design in which each participant was randomised to receive either propranolol or placebo in a double blind manner. Participants’ pulse rate was recorded using a pulse oximeter. Measures were obtained immediately prior to tablet administration, and at 30 min intervals thereafter. Additionally, participants’ mood was assessed using Visual Analogue scales (0–10 anchors) at three time points (before, 60 min after, and 150 min after propranolol/placebo administration) in which participants rated how sad, happy, alert, tired, angry or anxious they felt.

The moral decision task was presented on a computer using e-prime software 90 min after propranolol/placebo administration. The task included 20 hypothetical moral dilemmas, drawn from Koenigs et al. (2007) (see Supplementary materials for a full list of moral dilemmas). Of these, 15 were ‘personal’ (i.e., involving ‘up-close-and-personal’ harm) and 5 were ‘impersonal’ (based on Greene et al., 2001). Personal dilemmas were mostly ‘high conflict’ dilemmas (*N* = 13), on which there is no general consensus about the answer (as classified by Koenigs et al., 2007; see also Kahane and Shackel, 2010). Of the 15 personal dilemmas, 7 described scenarios where the victim of the proposed harmful act would inevitably die even if this act were not chosen (Huebner et al., 2011). All dilemmas were presented in random order.

Participants read the dilemmas on screen in their own time, and responded using the keypad. Participants were asked to rate how morally acceptable the action was on a 6 point scale (0, *completely morally unacceptable*; 5, *completely morally acceptable*), and whether they would themselves perform this action (0, *absolutely not*; 5, *absolutely yes*).

An example of a personal moral dilemma is *The Crying Baby* dilemma:

*Enemy soldiers have taken over your village. They have orders to kill all remaining civilians. You and some of your townspeople have sought refuge in the cellar of a large house. Outside you hear the voices of soldiers who have come to search the house for valuables. Your baby begins to cry loudly. You cover his mouth to block the sound. If you remove your hand from his mouth his crying will summon the attention of the soldiers who will kill you, your child, and the others hiding out in the cellar. To save yourself and the others you must smother your child to death.*

*How morally acceptable is it to smother your child in order to save yourself and the other townspeople?*

*Would you smother your child in order to save yourself and the other townspeople?*

Participants’ responses and their response times (measured at the onset of the question) were recorded. Participants also performed a task that measured implicit attitudes to other groups; the results of that investigation has been reported separately (Terbeck et al., 2012).

## 3 Results

*t*-Tests for independent sample revealed that the propranolol and placebo group did not differ in age, or body mass index (both *p*s > .05). Repeated measures analyses of variance (ANOVAs) for the six mood ratings from the visual analogue scales showed that there were no differences in reported mood between the propranolol and placebo group at any time (all *p* > .05).

**Fig. 1:**
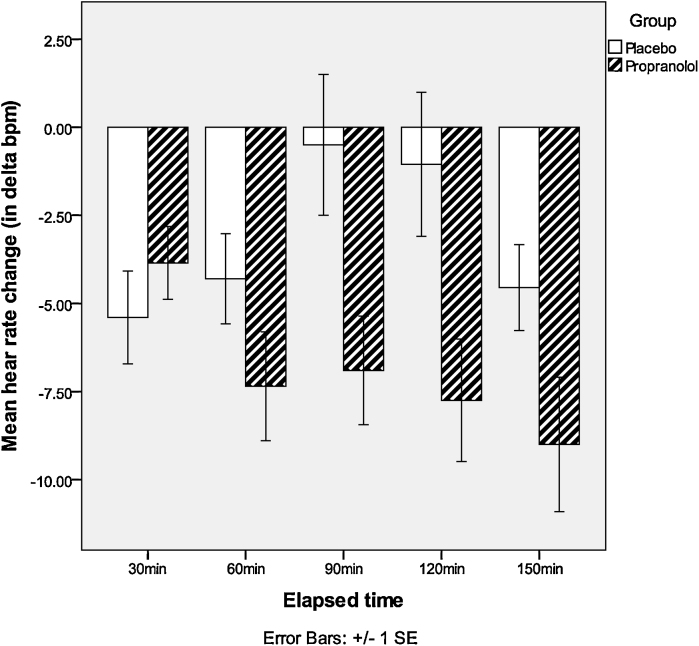
Heart rate change in propranolol and placebo groups.

A 2 treatment (propranolol vs. placebo) × 5 (times) repeated measures ANOVA was conducted to assess the heart rate change (difference in heart rate from the baseline recording). A significant interaction of time and group was found, *F*(4,35) = 9.27, *p* = .002. Post hoc *t*-tests showed that the decrease in heart rate was significantly greater in the propranolol as compared to the placebo group at 90 min, *t*(38) = −2.54, *p* = .02, and 120 min, *t*(38) = −2.50, *p* = .02, after treatment (see Fig. 1).

For moral judgement responses two different means (one for the 15 personal dilemmas and one for the 5 impersonal dilemmas) were calculated. The Kolmogorov–Smirnov test showed that the sample was not normally distributed (*p* < .05). Therefore data were log transformed, and 3 sub scores from the placebo group, which were detected as outliers, were not included. This led to optimal Kolmogorov–Smirnov tests for personal and impersonal dilemmas in placebo and propranolol group (all *p*s = .20).

**Fig. 2:**
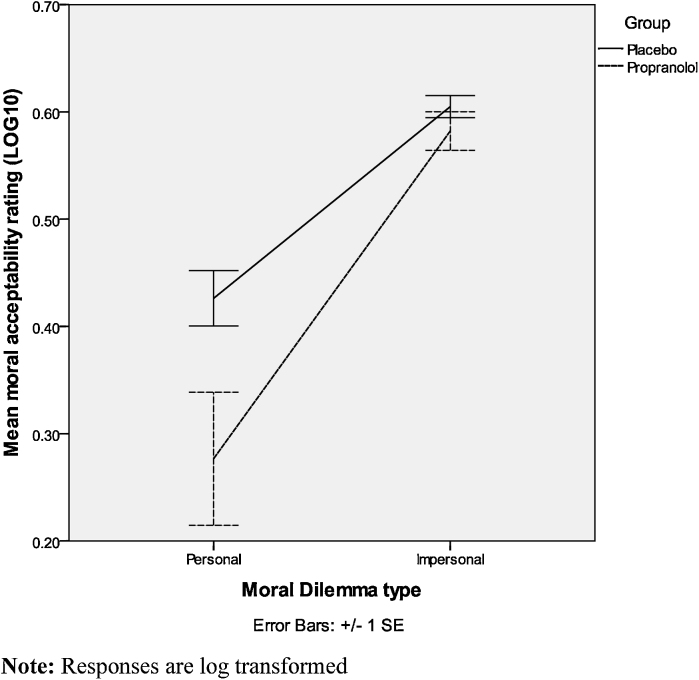
Mean moral acceptability ratings for personal and impersonal moral dilemmas in propranolol and placebo groups.

We conducted separate ANOVAs for the *moral acceptability* question (“Is the action morally acceptable?”) and for the *performance* question (“Would you perform the action?”). First a 2 (treatment: propranolol vs. placebo) × 2 (dilemma type: personal vs. impersonal) mixed ANOVA was conducted on the moral acceptability question. We found a main effect of dilemma type (*F*(1,35) = 123.39, *p* < .00). We also found a significant dilemma type × treatment interaction (*F*(1,35) = 5.20, *p* = .03). Individuals generally rated the proposed action in personal dilemmas (*M* = .36, *SD* = 23) as less morally acceptable than in impersonal dilemmas (*M* = .59, *SD* = .10), *t*(36) = −7.83, *p* < .00; *t*(39) = 12.00, *p* < .00. Importantly, participants in the propranolol group rated actions as less morally acceptable (*M* = .27, *SD* = .28) than did participants in the placebo group (*M* = .43, *SD* = .11) in personal, *t*(37) = 2.18, *p* = .04; *d* = .72, but not in impersonal, *t*(37) = 1.07, *p* = .29, moral dilemmas (see Fig. 2).

A 2 (treatment: propranolol vs. placebo) × 2 (dilemma type: personal vs. impersonal) mixed ANOVA was also performed on the performance question. This revealed a main effect of dilemma type, *F*(38,1) = 147.20, *p* < .00, but no interaction of main effect with treatment (all *p*s > .2). Overall individuals were more likely to rate that they would perform the action in impersonal (*M* = .56, *SD* = .07) as compared to personal (*M* = .25, *SD* = .20) moral dilemmas, *t*(39) = 12.00, *p* < .00. In addition there were no response time differences for the performance question.

As could be expected, there was a significant correlation between the answers to the moral acceptability question and the performance question (*r* = .53, *p* = .00). However, individuals generally rated the moral acceptability of the proposed act as higher than they rated their willingness to actually perform this act. This was the case both for personal, *t*(39) = 2.95, *p* = .01, and for impersonal, *t*(38) = 3.12, *p* = .00, moral dilemmas.

A previous study has reported a positive correlation between working memory capacity and utilitarian judgement in personal dilemmas (Moore et al., 2008), but only in dilemmas where the act endorsed (e.g., killing one person to save five others) would not make the person harmed worse off, because they would inevitably suffer this harm (because, for example, everyone would die if one does not act; cf. Huebner et al., 2011). Since some studies have suggested that propranolol can reduce working memory capacity (Chamberlain et al., 2006; Müller et al., 2005), we controlled for this possible influence by performing a 2 (treatment: propranolol vs. placebo) × 2 (dilemma type: inevitable vs. non-inevitable harm) mixed ANOVA. There was no main effect of or interaction involving dilemma type (all *p*s > .2), suggesting that the effect we observed was not moderated by working memory capacity. In addition, we found no effect or co-variance of gender with any of the observed effects (all *p*s > .2).

**Fig. 3:**
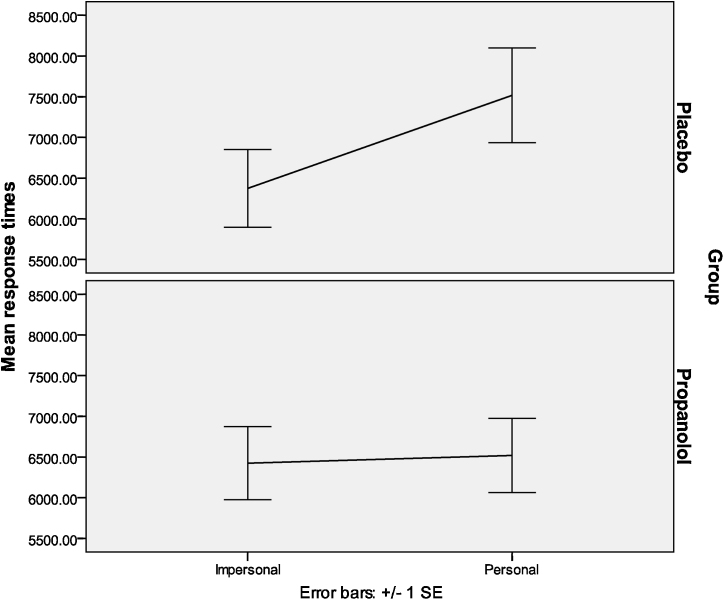
Response times for personal and impersonal moral dilemmas in both groups.

A 2 (treatment: propranolol vs. placebo) × 2 (dilemma type: personal vs. impersonal) mixed ANOVA was also computed for response times for the acceptability question. We found a significant main effect of dilemma type, *F*(1,38) = 4.65, *p* = .04. The dilemma type and group interaction was found to be significant (*F*(1,38) = 5.94, *p* = .02). Individuals in the placebo group took significantly longer to rate the moral acceptability of acts in personal (*M* = 7517.21, *SD* = 2596.02) as compared to impersonal (*M* = 6374.09, *SD* = 2139.90) dilemmas (*t*(19) = −2.58, *p* = .02). For the propranolol group the difference in response time for personal (*M* = 6519.6533, *SD* = 2041.58) and impersonal (*M* = 6424.61, *SD* = 2011.10) dilemmas was not significant (*p* > .05) (see Fig. 3). There were no group differences on the performance question (all *p*s > .2). In addition, we found no significant differences in response times between propranolol and placebo group in personal or impersonal dilemmas in the performance question (both *p*s > .05).

Finally, we investigated whether individuals in the propranolol group were more decisive in their judgement of moral acceptability. Scores of 2 or 3 on moral judgments were categorised as indecisive (i.e., neither clearly deontological nor utilitarian), and scores of 0 or 1 and 4 or 5 as decisive (i.e., clearly either deontological or utilitarian). On average, participants in the propranolol group made 29.3% indecisive judgments, compared to 40.7% in the placebo group. A Mann–Whitney *U*-test confirmed that individuals in the propranolol group made more decisive judgments (*U* = 123.00; *z* = −2.10, *p* = .04) in personal moral dilemmas.

## 4 Discussion

Suppression of noradrenergic-mediated arousal by propranolol was associated with a significant difference in moral judgement compared to placebo, suggesting a causal role for noradrenergic pathways in moral decision-making. Contrary to our hypothesis, subjects who took propranolol were more likely to make deontological judgments, and to reject contrary utilitarian solutions to moral dilemmas, but only when these utilitarian solutions involved ‘up close and personal’ harm to an innocent victim. Propranolol was also associated with reduced response times and increased ‘decisiveness’ compared to placebo. It was also associated with reduced heart rate, suggesting that the observed effects may be due to a general reduction in emotional arousal. In what follows we examine the implications of these findings for recent debates about the role of emotion in moral decision-making, and consider what causal mechanisms may explain our results.

Previous research has been taken to show that deontological judgments are largely driven by emotion (Greene, 2008). However, our results suggest that general emotional physiological arousal is not likely to play an essential role in generating deontological judgments. Propranolol in single doses is a well established technique for assessing the role of noradrenaline pathways in emotional responses (Cahill et al., 1994; Harmer et al., 2001; Hurlemann et al., 2010). Although we found no significant difference in self reported mood between the propranolol and placebo groups, heart rates in the propranolol group were significantly lower, suggesting a reduction in physiological arousal, in line with numerous previous studies (Hurlemann et al., 2010; Strange and Dolan, 2004; Van Stegeren et al., 2005). However, our results cannot rule out that deontological judgments nevertheless involve secondary emotions such as guilt, which may be based more on cognitive appraisal than on physiological arousal (Johnson-Laird and Oatley, 1992).

It is unclear at this stage why reduction in noradrenergic-mediated arousal would lead to an increase in deontological judgement. Rogers et al. (2004) reported that propranolol reduced the discrimination between large and small possible losses in a gambling task when the probability of winning was relatively low and the probability of losing was high. Since personal moral dilemmas can be understood as involving a choice between two options that involve highly probable (or even certain) loss of life, it might be suggested that participants under propranolol were exhibiting a similar lack of discrimination between differences in magnitude of overall loss. However, if propranolol generally reduced concern for consequences, it should affect responses to both personal and impersonal dilemmas, whereas we observed an increase in deontological judgement only in personal dilemmas.

It seems more likely that the observed effect was due to an increase in aversion to harming others, in line with extensive evidence that propranolol reduces aggression (Greendyke et al., 1984, 1986; Silver et al., 1999; Yudofsky et al., 1981). Interestingly, a decrease in aggression has also been associated with selective serotonin reuptake inhibitors such as citalopram (Armenteros and Lewis, 2002; Vartiainen et al., 1995), which have recently also been shown to increase deontological judgement (Crockett et al., 2010).

In line with this suggestion, propranolol influenced moral judgement only in ‘personal’ dilemmas, where in order to save more lives, one had to engage in an up close and personal act of direct violence against a single innocent person (Greene et al., 2001). This selective effect is in line with previous studies where psychophysiological interventions or neural damage affected moral judgement only in personal dilemmas or a ‘high conflict’ subset of such dilemmas (Crockett et al., 2010; Koenigs et al., 2007; Tassy et al., 2012; Youssef et al., 2012). Note that although there was a greater consensus in responses to ‘impersonal’ moral dilemmas which did not involve such direct harm (as indicated by lower standard deviation: .592 compared to .972), the selective effect was not due to a floor or ceiling effect in these impersonal dilemmas, given that we used a continuous (0–5 scale) rather than binary measure of moral permissibility, and permissibility ratings for both dilemma categories showed normally distributed data with *M* = 3.91 (impersonal) and *M* = 2.44 (personal).

It is intriguing that although propranolol increased rates of deontological judgement in personal dilemmas, it did not have a parallel affect on subjects’ willingness to perform the proposed act. This difference may, however, reflect a floor effect, given that most subjects were already generally averse to actually performing harmful acts even when they rated them morally acceptable.

Another intriguing result is the increase in decisiveness in moral judgement, coupled with a decrease in response time in personal dilemmas, compared to the placebo group. These findings are in line with previous research suggesting that deontological judgments in such dilemmas are based on an intuitive response (Cushman et al., 2006; Kahane et al., 2012). They suggest that subjects under propranolol engaged in less deliberation, and were more confident of their immediate responses than subjects who received a placebo. Although some studies have associated propranolol with reduced working memory capacity (Chamberlain et al., 2006; Müller et al., 2005), this is unlikely to explain this effect, since the only study so far to establish a direct relation between working memory capacity and utilitarian judgement found such a relation only in dilemmas where the personal harm was inevitable (Moore et al., 2008) and such a selective effect was not observed in the present study. It seems more likely that an increase in aversion to harming caused the deontological response to strongly dominate decision-making, leading both to an increase in deontological judgement and to faster and more decisive responses. In addition, it is possible that the reduction in anxiety that is associated with propranolol (Tyrer and Lader, 1974; Weatley, 1969) also played a role in increasing judgement confidence.

Propranolol operates on noradrenergic pathways both in the central and the peripheral nervous system. Further research using a peripherally acting beta-adrenoceptor antagonist is thus needed to determine whether the observed effect of propranolol on moral decision making is due to central beta-adrenoceptor blockade or to antagonist effects in the peripheral sympathetic nervous system. In addition, more sensitive measures of heart rate and other markers of physiological arousal are needed to investigate the temporal course of emotional arousal when individuals under propranolol are engaged with moral dilemmas.

Crockett et al. (2010) report that a single dose of the noradrenaline re-uptake inhibitor, atomoxetine, failed to influence moral judgement in a selection of personal moral dilemmas, whereas from our findings a decrease in deontological judgements might be expected. However, atomoxetine would be expected to facilitate neurotransmission at both alpha and beta adrenoceptors; hence its effect on moral decision-making might not be simply contrary to that of propranolol. It should also be noted that the Crockett et al. study employed a within group design while we used a between group method. While our design avoids the issue of learning effects, it is open to the objection that our findings may be confounded by baseline differences between the two groups. We tried to minimise this possibility by careful demographic matching of the treatment groups but it would be of interest to see if our findings can be replicated in a cross-over design.

Although further research is needed to clarify the exact role of noradrenergic arousal in moral judgement, our study provides strong evidence that propranolol, a widely used drug, can influence moral decision-making. Our finding is especially important in the context of the ongoing debate about the potential use of propranolol in the prevention or treatment of post-traumatic stress disorder in military or rescue service personnel (Donovan, 2010; Henry et al., 2007), given that the hypothetical dilemmas used in the present study involve such difficult decisions in extreme emergency situations.
