# Supplementary material for: Beta adrenergic blockade reduces utilitarian judgement
Source: Biol Psychol. 2013 Feb;92(2):323–8. doi: 10.1016/j.biopsycho.2012.09.005 (PMC3573226; doi:10.1016/j.biopsycho.2012.09.005)
Supplement: Supplementary file 1 [file mmc1.pdf]

SUPPLEMENTARY MATERIAL

for

Beta Adrenergic Blockade Reduces Utilitarian Judgment

Terbeck, Sylvia*; Kahane, Guy; McTavish, Sarah; Savulescu, Julian; Levy, Neil; Hewstone, Miles; Cowen, Philip J.

* To whom correspondence should be addressed:

sylvia.terbeck@psy.ox.ac.uk

Moral Dilemmas

The moral dilemmas were drawn from Koenigs et al. (2007). Below we list the titles of impersonal and personal dilemmas used. See Koenigs et al. (2007) for the full text of each dilemma, and for the criteria for classifying scenarios as low- vs. high-conflict.

<u>Impersonal Moral Scenarios</u>

1. Standard Trolley
2. Standard Fumes
3. Vaccine Policy
4. Speedboat
5. Guarded Speedboat

<u>Personal Moral Scenarios</u>

1.	Transplant	*Low-conflict*
2.	Footbridge	*High-conflict*
3.	Crying Baby	*High-conflict / death inevitable*
4.	Plane Crash	*Low-conflict/ death inevitable*
5.	Lifeboat 2	*High-conflict/ death inevitable*
6.	Preventing the Spread 2	*High-conflict*
7.	Ecologists	*High-conflict/ death inevitable*
8.	Bomb 2	*High-conflict*
9.	Submarine	*High-conflict/ death inevitable*
10.	Lawrence of Arabia	*High-conflict*
11.	Sophie's Choice	*High-conflict/ death inevitable*
12.	Sacrifice Mean	*High-conflict/ death inevitable*
13.	Vitamins	*High-conflict*
14.	Vaccine Test	*High-conflict*
15.	Euthanasia	*High-conflict*

References

Koenigs, M., Young, L., Adolphs, R., Tranel, D., Cushman, F., Hauser, M., Damasio, A. R. (2007) Damage to the prefrontal cortex increases utilitarian moral judgments. *Nature*, 446, 908-911.
